# Psychopathology of Couple Relationship: A Moderation Analysis on Love Addiction

**DOI:** 10.1002/ijop.70079

**Published:** 2025-07-16

**Authors:** Roberta Gabriella Cavalli, Patrizia Velotti

**Affiliations:** ^1^ Department of Dynamic and Clinical Psychology, and Health Studies, Faculty of Medicine and Psychology Sapienza University of Rome Rome Italy

**Keywords:** attachment, couple relationship, dyadic emotion regulation, love addiction, psychopathology

## Abstract

In recent decades, a new clinical condition has been proposed as a potential psychopathological disorder warranting further investigation. Love addiction (LA) is characterised by an overwhelming investment in romantic relationships, resulting in excessive dependence on the partner, expressed through obsessive behaviours and beliefs. Although current literature suggests that LA may best fit within the behavioural addictions diagnostic cluster, several authors emphasise the relational nature of the disorder. We conducted two moderation analyses to examine associations with LA. In Study 1, we tested the moderation role of attachment (ECR‐SF) in the relationship between obsessive‐compulsive disorder (ROCI) and LA (assessed by the LAI). In Study 2, we explored the moderating role of dyadic emotion dysregulation (DDY) in the relationship between self‐defeating tendencies (SELF‐DISS) and LA. Study 1 involved a sample of 720 adults, and Study 2 included 672 adults. A significant and positive interaction was found between anxious attachment and relational obsessive‐compulsive disorder (Study 1) as well as between self‐defeating and dyadic emotion dysregulation (Study 2) in their associations with Love Addiction. These preliminary findings contribute to the current understanding of Love Addiction, highlighting the significant role of relational dimensions in its development.

## 1 Introduction

According to Shoop (2019), initiating and establishing romantic relationships represent one of the key milestones of human experience. Couple bonds form one of the pillars underlying psychophysical health, and the quality of these bonds is a crucial source of individual well‐being (DeWall et al. 2011). In a review conducted by Gómez‐López et al. (2019) found that higher levels of marital intimacy and satisfaction are associated with higher levels of self‐esteem, a more positive self‐concept, higher life satisfaction, and overall well‐being. Conversely, marital distress can serve as a risk factor for individual psychopathology, including depression, anxiety, and suicide (Kazan et al. 2016), as well as for dysfunctional relationships characterised by violence, relational obsessive‐compulsive disorder, and unrealistic relational beliefs (Velotti et al. 2022).

## 2 Understanding Couple Relationships: Attachment and Emotion Regulation

Since Hazan and Shaver's pioneering study (1987), researchers have increasingly focused on attachment bonds in adulthood. From this perspective, the psychological functions originally fulfilled by the primary caregiver are transferred to romantic partners, who become new attachment figures, ensuring that individuals' attachment needs are met. In adult attachment theory, bonds develop along two dimensions: avoidance of intimacy, aimed at maintaining independence, and abandonment anxiety, aimed at maintaining closeness with significant others (Shaver et al. 2019). Attachment theory provides a robust framework for understanding the dynamics of intimate relationships (Velotti et al. 2023). Numerous studies have demonstrated a relationship between attachment insecurity and relational outcomes, including higher levels of conflict, lower satisfaction, diminished support, and reduced connectedness (Candel and Turliuc 2019; Murphy et al. 2022). Moreover, research within the attachment framework has proven fruitful in elucidating mechanisms underlying the development and maintenance of pathological conditions. Two recent meta‐analyses (Velotti et al. 2022; Cataudella et al. 2023) have highlighted the role of anxiety and avoidance in violent relationships, from both victims' and perpetrators perspectives. The influence of attachment styles has also been confirmed in psychopathologies related to intimate relationships, such as relational obsessive‐compulsive disorder (ROCD). According to Kabiri et al. (2017) and Trak and Inozu (2019), attachment anxiety has been associated with more severe ROCD, representing a vulnerability factor in the disorder's development.

Closely related to attachment theory is the construct of emotion regulation (ER), defined as the ability to influence the intensity, duration, and quality of experienced emotions to meet environmental demands (Thompson 1994). Difficulties in this process—encompassing emotional acceptance, clarity, understanding, or control—are termed emotion dysregulation (ED; Gratz and Roemer 2004). Recently, there has been a growing empirical and theoretical focus on the interpersonal and dyadic processes underpinning emotion regulation. Faccini et al. (2023) identified couple relationships as an optimal context for conceptualising the dyadic nature of emotion regulation, introducing the construct of dyadic emotion dysregulation, which refers to difficulties in regulating emotions at an interpersonal level and in response to the other person's emotional states.

As noted, during the transition to adulthood, several functions initially acquired in early bonds are transferred to romantic relationships, including dyadic emotion regulation capacities (Faccini et al. 2023). Within a protective environment, early caregiver–child interactions foster social synchronicity, laying the foundation for the ability to recognise and regulate one's own and others' emotions. The parallel development of attachment and emotion regulation from childhood to adulthood supports the view of the attachment system as an emotion regulation system, with regulatory capacities extending from internalised relational models (Fávero et al. 2021).

Thus, emotion regulation can be recognised as a transdiagnostic construct underpinning both individual psychopathology and relational functioning. The ability to effectively regulate emotions within the dynamics of a couple relationships has been associated with greater emotional satisfaction and relationship stability. Conversely, deficits in emotion regulation are linked to lower marital satisfaction, reduced intimacy, a higher risk of separation, increased dependency, and controlling behaviours (Rick et al. 2017; Fávero et al. 2021).

## 3 A New and Unclear Clinical Picture: Love Addiction

Given the influence of couple bonds on individual mental health (DeWall et al. 2011), it is essential to consider dysfunctional conditions that may lead to distress. Recently, several authors and researchers have identified excessive dependence on one's partner as a potential psychopathological condition warranting further investigation. Love Addiction is characterised by an overwhelming investment in a romantic relationship, resulting in excessive dependence on the partner, expressed through obsessive behaviours and beliefs (Reynaud et al. 2010; Earp et al. 2017; Redcay and Simonetti 2018). Since its introduction into the literature, a major challenge concerning the operationalisation of LA has been its classification within the diagnostic cluster of behavioural addictions. In this context, the component model proposed by Griffiths (2005) has shown promise in identifying various forms of behavioural addictions. However, many theoretical contributions (Sanches and John 2018; Redcay and McMahon 2021) argue that the core of LA lies in the relationship with the significant other, emphasising its relational nature and distinguishing it from other forms of addiction.

Recently, some authors (Cavalli et al. 2024) have proposed examining the construct LA within a theoretical framework alternative to the addiction model (Griffiths 2005). Given the dynamics of dependence and the search for a partner as a regulator of affective states, attachment theory offers a solid foundation for understanding this construct.

Secure attachment allows for flexibility between partners in both providing and seeking care during times of distress. In contrast, insecure attachment in romantic relationships leads to a lack of trust in the partner's availability, prompting alternative strategies to maintain closeness (Mikulincer and Shaver 2019). From this perspective, the balance between dependence and independence becomes disrupted, and individuals with insecure attachment styles tend to adopt rigid patterns, resulting in an unhealthy form of dependence characterised by emotional inflexibility. In anxious attachment, individuals remain in a constant state of dependence on their partner due to negative expectations regarding the availability of significant others. In avoidant attachment, distrust in others and fear of excessive emotional closeness foster an extreme need for independence (Velotti et al. 2011; Mikulincer and Shaver 2019). Considering the role of dependence in romantic relationships and the ability to be both caregiver and care receiver, attachment theory provides a valuable framework for understanding partner dependence, particularly in anxious attachment, where excessive reliance on the other is evident.

The threat of losing a partner is behaviorally associated with efforts to avoid abandonment and emotionally linked to the emergence of anxiety and stress (Sanches and John 2018). Sussman (2010) describes the ‘saviour’ role often attributed to romantic relationships in overcoming adversity, noting the presence of magical thinking and obsessive tendencies in individuals with love addiction. More recently, Atkinson and Vernon (2017) introduced the concept of the self‐defeating interpersonal style, referring to a chronic tendency to tolerate mistreatment in romantic relationships. This pattern involves overlooking the negative consequences of a relationship (e.g., repeated mistreatment) in favour of pursuing immediate or psychologically essential goals, such as the fulfilment of emotional needs. In line with this, Arbinaga et al. (2021) describe the inability of individuals with love addiction to recognise and end relationships that cause them suffering. These theoretical premises suggest not only highlight the need to deepen understanding of the mechanisms underlying LA but also support the impotence of framing these investigations within a relational perspective that considers Love Addiction among possible relational psychopathologies.

## 4 The Current Study

Based on these considerations, the relationship with the significant other takes precedence in the life of the individuals with LA, emphasising the need to examine the relational nature of the construct. The present studies aim to investigate the psychopathological mechanisms underlying the onset of LA within the theoretical framework of attachment theory and emotion regulation.

### 4.1 Study 1: Rational and Hypothesis

As previously mentioned, the link between attachment insecurity and dysfunctional relationships has been widely demonstrated (Murphy et al. 2022; Cataudella et al. 2023). Additionally, theorists of LA (Reynaud et al. 2010) have noted significant overlap between individuals with anxious attachment and those with LA. Features such as fear of abandonment and rejection, efforts to restore closeness, and an excessive need for approval are common to both conditions, suggesting a possible role of attachment insecurity in the development of Love Addiction. Beyond the characteristics shared with anxious (preoccupied) attachment, Briggie and Briggie (2015) and Sussman (2010) also identified the presence of magical thinking, persistent doubts about the relationship, and questioning the partner's interest—elements similarly observed in Relational Obsessive‐Compulsive Disorder (ROCD; Melli et al. 2018). In the conceptualisation of ROCD as well, the role of anxious attachment has been emphasised (Trak and Inozu 2019). When faced with the threat of abandonment, anxiously attached individuals tend to adopt less adaptive coping strategies, making them more vulnerable to developing obsessive concerns about the relationship (Kabiri et al. 2017). Given the conceptual overlap between ROCD and LA and the influence of anxious attachment in both conditions, Study 1 aims to provide empirical data to support this theoretical formulation. Clarifying the association among LA, ROCD, and anxious attachment may help position LA within the framework of couple‐related psychopathology, thereby distinguishing it more clearly from the traditional behavioural addiction model (Griffiths 2005).

It was hypothesised that:

*A positive and significant association between Love Addiction, relational obsessive‐compulsive disorder, and anxious attachment*.

*The association between relational obsessive‐compulsive disorder and Love Addiction would be stronger at higher levels of anxious attachment*.

### 4.2 Study 2: Rational and Hypothesis

According to Arbinaga et al. (2021) and Reynaud et al. (2010), one of the pathological features of Love Addiction (LA) is the inability to end a relationship, even when it causes significant suffering. A similar mechanism has been identified in abusive relationships, where it acts as a barrier to reporting violence. The motivations behind this difficulty may vary and include lack of external support, hope that the partner will change, fear of abandonment, or a belief in deserving the abuse (Ambler et al. 2023). Atkinson and Vernon (2017) proposed the concept of self‐defeating interpersonal style, conceptualised as a relational mode characterised by a negative self‐model, insecure attachment, and a tendency to accept various forms of maltreatment. In this sense, such a style could help predict over‐dependence on the romantic partner.

As previously noted, the theoretical framework of emotion regulation has proved particularly useful in understanding the mechanisms underlying the phenomenon of IPV, both from the perpetrators' (Velotti et al. 2022) and the victims' (Muñoz‐Rivas et al. 2021) perspectives. The study by Muñoz‐Rivas et al. (2021) not only revealed high levels of emotion dysregulation among IPV victims but also found a link between dysregulation and PTSD severity, suggesting that emotion dysregulation plays a critical role in the deterioration of victims' mental health. Moreover, difficulties in emotion regulation represent a transdiagnostic factor implicated in various addictions (Gioia et al. 2021) and in reduced marital quality (Fávero et al. 2021). Given the purposes of the present research, it was decided to investigate whether dyadic emotion dysregulation could offer a comprehensive understanding of love addiction, considering the dyadic nature of the romantic relationships (Faccini et al. 2023).

Based on these considerations, in Study 2, it was hypothesised that:

*A positive and significant association between Love Addiction, self‐defeating interpersonal style, and dyadic emotion dysregulation*.

*The association between self‐defeating interpersonal style and Love Addiction would be stronger at higher levels of dyadic emotion dysregulation*.

## 5 Study 1

### 5.1 Method

#### 5.1.1 Participants and Procedure

A total of 720 Italian adults were recruited, comprising individuals from the general population as well as participants diagnosed with Love Addiction. A convenience sampling method was employed, using an online survey comprising a battery of questionnaires. For the recruitment of the clinical subsample, collaborations were established with mental health professionals who identified and referred individuals with a diagnosis of LA. The inclusion criteria were: (1) being of legal age and (2) having sufficient language comprehension to complete the questionnaires. The sample size was determined based on a power analysis conducted using G*Power 3.1.9.6 software, with an a priori analysis tailored to moderation analyses and the number of variables (including covariates). The mean age of participants was 33.43 (SD = 13.07), with an age range of 18–84. 72.6% of the sample were females (*n* = 523), 26.9% were males (*n* = 194) and 0.4% selected ‘other’ (*n* = 3). Data were collected through self‐report measures administered via the Eusurvey platform. Prior to participation, the study objectives and procedures were explained, and informed consent was obtained from all participants. The research protocol was approved by the Ethics Committee of the Sapienza University of Rome (N. 0078/22).

#### 5.1.2 Measures

*Demographic information* was collected through an ad‐hoc self‐report questionnaire. Participants were asked to provide information about age, gender, education level, socioeconomic status, and place of residence.

The *love addiction inventory* (LAI; Costa et al. 2021) is a 24‐item self‐report that measures Love Addiction according to Griffiths' component model (2005) corresponding to the instrument subscales: salience, tolerance, mood modification, relapse, withdrawal, and conflict. A total score of love addiction can be achieved from the sum of subscale scores. Psychometric properties of LAI were adequate with a Cronbach's alpha range of 0.76–0.95 (Costa et al. 2021).

The *experiences in close relationship‐short* (ECR‐S; Wei et al. 2007) is a 12‐item questionnaire that measures adult attachment dimensions, anxiety, and avoidance. In the original study (Wei et al. 2007), ECR‐S showed good internal consistency with a range of 0.77–0.86 for anxiety and a range of 0.78–0.88 for avoidance.

The *relationship obsessive‐compulsive inventory* (ROCI; Doron et al. 2012; Melli et al. 2018) is a 12‐item instrument that measures relational OCD according to three dimensions: Love for Partner, Adequacy of Relationship, and Partner's love. The questionnaire provides a total score of relationship obsessive‐compulsive. In its Italian validation (Melli et al. 2018), the internal consistency was reached with an alpha range of 0.81–0.93.

#### 5.1.3 Statistical Analyses

First, frequencies, means, and standard deviation of demographic information and variables were computed. The internal consistency of the instruments was assessed using Cronbach's alpha values. To examine the associations between the investigated variables, a partial Pearson correlation was computed, controlling for age and gender. Finally, moderation analyses were conducted using Model 1 of the PROCESS macro (Hayes 2022). All statistical analyses were performed using IBM SPSS Statistics for Windows, version 27.

### 5.2 Results

#### 5.2.1 Descriptive Analyses

Psychometric properties of the used instrument, including internal consistency, means, and standard deviations, are presented in Table 1.

**TABLE 1:** Description of variables involved in Study 1.

		M	SD	*α*
LAI	Total score	59.31	15.60	0.93
	Salience	12.88	3.50	0.94
	Withdrawal	7.91	3.40	0.88
	Tolerance	11.26	3.96	0.92
	Mood modification	11.56	4.01	0.89
	Relapse	7.71	3.69	0.89
	Conflict	7.96	3.60	0.89
ECR‐SF	Anxiety	20.95	7.15	0.70
ROCI	Total score	11.92	10.05	0.93
	Love for partner	3.13	3.51	0.88
	Adequacy of relationship	4.46	3.88	0.87
	Partner's love	4.32	3.83	0.84

Abbreviations: ECR‐SF, experience in close relationships short‐form; LAI, love addiction inventory; ROCI, relationship obsessive‐compulsive inventory.

#### 5.2.2 Partial Correlations Between Variables

As displayed in Table 2, our first hypothesis was confirmed. ECR‐S anxiety positively and significantly correlated with LAI and its subscales (*r* = 0.24–0.47, *p* < 0.01). Positive and significant associations were also observed between ROCI (*r* = 0.11–0.46, *p* < 0.01) and LAI. However, no significant associations were found between LAI salience and mood modification and ROCI love for partner (*p* = 0.757; *p* = 0.317) or ROCI adequacy of relationship (*p* = 0.065; *p* = 0.079, respectively). Lastly, all ROCI subscales were positively correlated with ECR‐SF anxiety (*r* = 0.33–0.60, *p* < 0.01).

**TABLE 2:** Partial correlations, controlling for age and gender in Study 1.

	1	2	3	4	5	6	7	8	9	10	11	12
1. LAI	—											
2. LAI salience	0.67**	—										
3. LAI withdrawal	0.77**	0.38**	—									
4. LAI tolerance	0.75**	0.50**	0.50**	—								
5. LAI mood modification	0.67**	0.47**	0.38**	0.45**	—							
6. LAI relapse	0.70**	0.29**	0.53**	0.32**	0.30**	—						
7. LAI conflict	0.65**	0.22**	0.50**	0.34**	0.20**	0.53**	—					
8. ECR‐SF anxiety	0.46**	0.26**	0.47**	0.35**	0.24**	0.34**	0.31**	—				
9. ROCI	0.41**	0.11**	0.46**	0.26**	0.11**	0.40**	0.40**	0.54**	—			
10. ROCI love for partner	0.28**	0.01	0.35**	0.14**	0.04	0.32**	0.35**	0.33**	0.87**	—		
11. ROCI adequacy of relationship	0.36**	0.07	0.41**	0.24**	0.07	0.38**	0.37**	0.51**	0.95**	0.78**	—	
12. ROCI Partner's love	0.45**	0.20**	0.48**	0.32**	0.18**	0.38**	0.35**	0.60**	0.86**	0.55**	0.73**	—

*Note:* **p < 0.01, *p < 0.05.

Abbreviations: ECR‐SF, experience in close relationship‐short form; LAI, love addiction inventory; ROCI, relationship obsessive‐compulsive inventory.

#### 5.2.3 Moderating Role of Attachment Anxiety in the Relationship Between ROCD and Love Addiction

**FIGURE 1:**
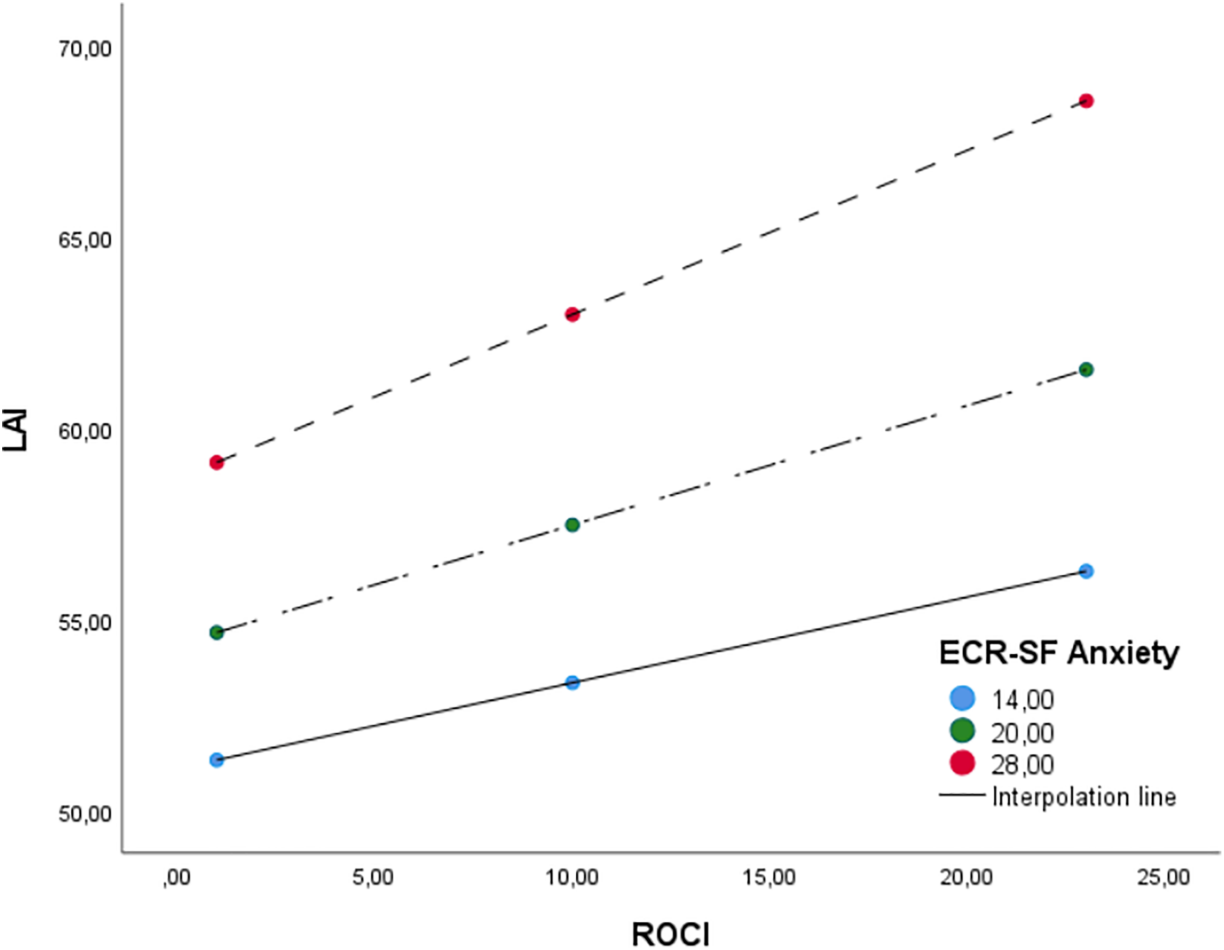
Slope analysis in Study 1.

To test the moderating role of ECR‐S Anxiety in the relationship between LAI and ROCI, we conducted a moderation analysis, entering age and gender as covariates. As shown in Table 3, the overall model was significant, *F* (5, 714) = 54.354, *R*2 = 0.27, *p* < 0.0001. The result indicated a non‐significant effect of ROCI (*b* = 0.01, *t* = 0.12, *p* = 0.900), but a significant effect of ECR‐SF anxiety (*b* = 0.54, *t* = 4.45, *p* < 0.0001). Furthermore, the interaction between ROCI and ECR‐SF Anxiety was significant and positive (*b* = 0.01, *t* = 2.36, *p* < 0.05) in relation to LA. Concerning covariates, sex was not significant (*b* = −1.11, *t* = −0.99, *p* = 0.320) while age was significant (*b* = −0.09, *t* = −2.40, *p* < 0.05). Lastly, slope analysis evidenced that ROCD was positively associated with Love Addiction when combined with attachment anxiety when scores are low (*b* = 0.22, *t* = 2.80, *p* < 0.01), moderate (*b* = 0.31, *t* = 5.04, *p* = < 0.00001), or high (*b* = 0.43, *t* = 6.25, *p* = < 0.00001) (see Figure 1), confirming the second hypothesis.

**TABLE 3:** Moderating effect of ROCI and ECRS‐anxiety in predicting LAI scores.

	*β*	SE	*t*	*p*
Constant	47.46	2.85	16.64	< 0.00001
Age	−0.09	0.03	−2.40	< 0.05
Sex	−1.11	1.11	−0.99	0.320
ROCI	0.02	0.15	0.12	0.900
ECR‐SF anxiety	0.54	0.12	4.45	< 0.00001
ROCI * ECR‐SF anxiety	0.01	0.00	2.36	< 0.05

*Note: R* ^2^ = 0.27; *p* < 0.00001.

Abbreviations: ECR‐SF, experience in close relationship‐short form; LAI, love addiction inventory; ROCI, relationship obsessive‐compulsive inventory.

## 6 Study 2

### 6.1 Method

#### 6.1.1 Participants and Procedure

A total of 672 Italian adults were recruited, including individuals from the general population as well as those diagnosed with LA. A survey containing a battery of questionnaires was used to recruit a convenience sample. For the clinical sample, collaborations with mental health professionals were established to identify and recruit patients diagnosed with LA. The inclusion criteria were: (1) being of legal age and (2) having sufficient language comprehension to complete the questionnaires. The sample size was determined based on a power analysis conducted using G*Power 3.1.9.6 software, with an a priori analysis considering the moderation analyses and the number of variables (including covariates). The mean age was 33.04 (SD = 12.95), with a range from 18 to 84 years. The sample comprised 71.6% females (*n* = 481), 28% males (*n* = 188), and 0.4% identified as ‘other’ (*n* = 3). Data were collected through a battery of self‐report measures available on the EUSurvey platform. Prior to participation, the study objectives and procedures were explained to the participants, and informed consent was obtained from all individuals. The study procedure was approved by the Ethical Board of Sapienza University of Rome (N. 0078/22).

#### 6.1.2 Measures

*Demographic information*: as mentioned in Study 1, demographic information was collected through an ad‐hoc questionnaire.

The *love addiction inventory* (LAI; Costa et al. 2021) is described in Study 1.

The *difficulties in emotion regulation scale dyadic* (DDY; Faccini et al. 2023): It adapts the difficulties in emotion regulation scale (Gratz and Roemer 2004) in the dyadic context. It comprises 12 items and two subscales: lack of dyadic awareness and lack of dyadic clarity. In the original study, the psychometric properties of the instrument were reached through an alpha of 0.84 for awareness and 0.83 for clarity.

The *self‐defeating interpersonal style scale* (SELF‐DISS; Atkinson and Vernon 2017) is a 35‐item questionnaire that assesses the tendency to enter tolerance‐battering relationships. The SELF‐DISS is composed of three subscales: insecure attachment, underserving self‐image, and self‐sacrificing nature, and a total score. In the original study, SELF‐DISS showed good reliability with an alpha range of 0.87–0.97.

#### 6.1.3 Statistical Analyses

To test the psychometric properties of each instrument, Cronbach's alpha was computed. Descriptive analyses were conducted, calculating frequencies, means, and standard deviations for demographic information and variables. To examine the association between the investigated variables, Pearson's r partial correlation was performed, controlling for age and gender. Moderation analyses were conducted using Model 1 of the PROCESS Macro (Hayes 2022). All statistical analyses were performed using statistical software for social science (SPSS) for Windows v.27.

### 6.2 Results

#### 6.2.1 Descriptive Analyses

Cronbach's alpha means and standard deviations of tested variables are displayed in Table 4.

**TABLE 4:** Description of variables involved in Study 2.

		M	SD	*α*
LAI	Total score	59.36	15.76	0.93
	Salience	12.91	3.52	0.95
	Withdrawal	7.87	3.37	0.88
	Tolerance	11.23	3.99	0.92
	Mood modification	11.63	4.02	0.90
	Relapse	7.75	3.67	0.89
	Conflict	7.94	3.58	0.89
DDY	Lack of dyadic awareness	11.02	4.09	0.84
	Lack of dyadic clarity	11.33	4.07	0.85
SELF‐DISS	Total score	77.60	23.67	0.92
	Insecure attachment	34.27	11.59	0.92
	Underserving self‐image	20.46	7.40	0.87
	Self‐sacrificing nature	22.86	8.88	0.88

Abbreviations: DDY, difficulties in emotion regulation scale dyadic; LAI, love addiction inventory; SELF‐DISS, self‐defeating interpersonal style scale.

#### 6.2.2 Partial Correlations Between Variables

To test the association between variables, Pearson's r was computed (see Table 5). Results indicated that lack of dyadic awareness was negatively associated with LAI salience and mood modification (*r* = −0.20 and *r* = −0.16, *p* < 0.01, respectively), as well as with lack of dyadic clarity (*r* = −0.07 and *r* = −0.07, *p* < 0.05, respectively), as assessed with DDY. Furthermore, lack of dyadic awareness was not associated with the LAI total score (*p* = 0.255) or LAI tolerance (*p* = 0.865). Dyadic emotion dysregulation was positively associated with all subscales of the LAI, including the total score (*r* = 0.19–0.32, *p* < 0.01). A negative and significant correlation was found between the SELF‐DISS total score and LAI mood modification (*r* = −0.18, *p* < 0.01). However, no association was observed between the SELF‐DISS underserving self‐image subscale and LAI salience (*p* = 0.056), partially confirming our third hypothesis. All other subscales of the SELF‐DISS were positively associated with the LAI and its subscales (*r* = 0.08–0.46, *p* < 0.01). Finally, a positive association was found between the SELF‐DISS and the DDY (*r* = 0.21–0.42, *p* < 0.01).

**TABLE 5:** Partial correlations, controlling for age and gender in Study 2.

	1	2	3	4	5	6	7	8	9	10	11	12	13
1. LAI	—												
2. LAI salience	0.69**	—											
3. LAI withdrawal	0.77**	0.40**	—										
4. LAI tolerance	0.76**	0.50**	0.50**	—									
5. LAI mood modification	0.69**	0.48**	0.40**	0.49**	—								
6. LAI relapse	0.71**	0.33**	0.55**	0.34**	0.32**	—							
7. LAI conflict	0.65**	0.22**	0.49**	0.35**	0.22**	0.53**	—						
8. DDY lack of dyadic awareness	0.04	−0.20**	0.19**	−0.00	−0.16**	0.19**	0.19**	—					
9. DDY lack of dyadic clarity	0.20**	−0.07*	0.30**	0.11**	−0.07*	0.32**	0.30**	0.60**	—				
10. SELF‐DISS	0.40**	0.14**	0.44**	0.26**	−18**	0.37**	0.33**	0.26**	0.42**	—			
11. SELF‐DISS insecure attachment	0.44**	0.19**	0.46**	0.29**	0.24**	0.40**	0.31**	0.23**	0.38**	0.88**	—		
12. SELF‐DISS underserving self‐image	0.25**	0.06	0.30**	0.11**	0.10*	0.26**	0.24**	0.21**	0.33**	0.80**	0.52**	—	
13. SELF‐DISS self‐sacrificing nature	0.28**	0.08*	0.32**	0.21**	0.08*	0.25**	0.28**	0.22**	0.36**	0.86**	0.60**	0.60**	—

*Note:* **p < 0.01, *p < 0.05.

Abbreviations: DDY, difficulties in emotion regulation scale dyadic; LAI, love addiction inventory; SELF‐DISS, self‐defeating interpersonal style scale.

#### 6.2.3 The Moderating Role of Dyadic Emotion Dysregulation in the Relationship Between Self‐Defeating and Love Addiction

**FIGURE 2:**
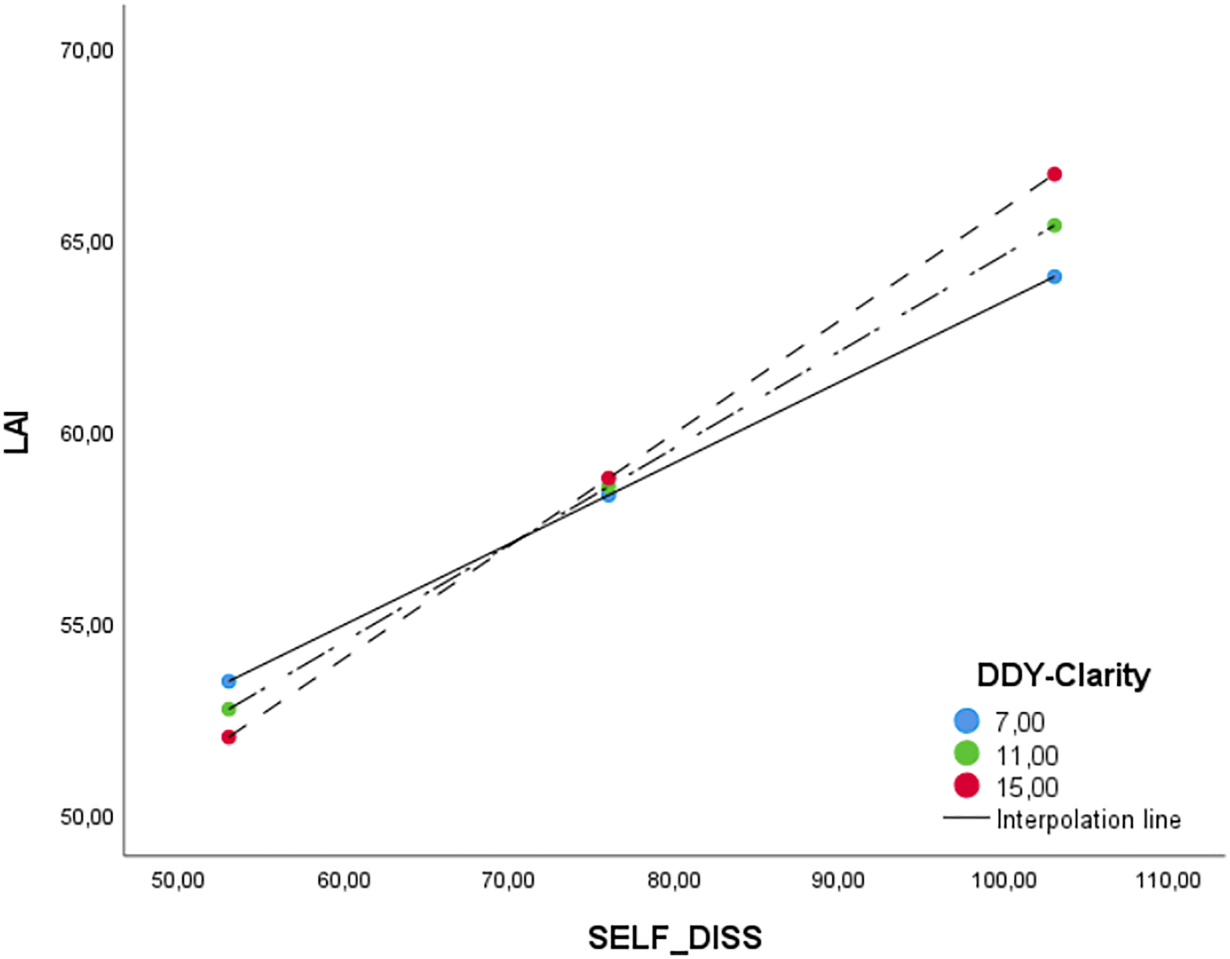
Slope analysis in Study 2.

Lastly, we tested the moderation role of DDY in the relationship between LAI and SELF‐DISS, competing age and gender as covariates. Consistent with the above results, no moderation effect was found for the interaction between SELF‐DISS and DDY lack of dyadic awareness (*b* = 0.00, *t* = 0.00, *p* = 0.993) (see Table 6). Instead, a significant and positive moderation effect between SELF‐DISS and DDY lack of dyadic clarity in relation to LA was observed (*b* = 0.01, *t* = 1.97, *p* < 0.05). As illustrated in Table 7, the result showed a significant effect of SELF‐DISS (*b* = 0.13, *t* = 2.16, *p* < 0.05) and a non‐significant effect of DDY lack of dyadic clarity (*b* = −0.73, *t* = −1.53, *p* = 0.124) on LA while the overall model was significant, *F* (5, 666) = 29.525, *R*2 = 0.18, *p* < 0.0001. Concerning covariates, sex and age were not significant (*b* = −1.48, *t* = −1.22, *p* = 0.222) and (*b* = −0.08, *t* = −1.90, *p* = 0.057) singly. Slope analysis evidenced that SELF‐DISS was positively associated with love addiction when combined with lack of dyadic clarity when scores were low (*b* = 0.03, *t* = 6.19, *p* < 0.00001), moderate (*b* = 0.02, *t* = 9.64, *p* = < 0.00001), or high (*b* = 0.03, *t* = 8.93, *p* = < 0.00001) (see Figure 2), confirming partially the fourth hypothesis.

**TABLE 6:** Moderating effect of SELF‐DISS and DDY lack of dyadic awareness in predicting LAI scores.

	*β*	SE	*t*	*p*
Constant	43.77	5.58	7.83	< 0.00001
Age	−0.07	0.04	−1.50	0.133
Sex	−1.33	1.21	−1.09	0.272
SELF‐DISS	0.28	0.07	3.98	< 0.001
DDY lack of dyadic awareness	−0.25	0.50	−0.49	0.623
SELF‐DISS * DDY lack of dyadic awareness	−0.00	0.00	−0.00	0.992

*Note: R* ^2^ = 0.18; *p* < 0.00001.

Abbreviations: DDY, difficulties in emotion regulation scale dyadic; LAI, love addiction inventory; SELF‐DISS, self‐defeating interpersonal style scale.

**TABLE 7:** Moderating effect of SELF‐DISS and DDY lack of dyadic clarity in predicting LAI scores.

	*β*	SE	*t*	*p*
Constant	51.22	5.46	9.37	< 0.00001
Age	−0.08	0.04	−1.90	0.056
Sex	−1.48	1.21	−1.22	0.221
SELF‐DISS	0.14	0.06	2.16	< 0.05
DDY lack of dyadic clarity	−0.72	0.47	−1.53	0.124
SELF‐DISS * DDY lack of dyadic clarity	0.01	0.00	1.97	< 0.05

*Note: R* ^2^ = 0.18; *p* < 0.00001.

Abbreviations: DDY, difficulties in emotion regulation scale dyadic; LAI, love addiction inventory; SELF‐DISS, self‐defeating interpersonal style scale.

## 7 Discussion

A substantial branch of research in psychology has highlighted the impact of the quality of romantic bonds on individuals' mental health (DeWall et al. 2011). Two theoretical frameworks that have proven particularly useful in understanding the complex mechanisms involved in intimate relationships are attachment theory and emotion regulation. Studies examining adult attachment have shed light on how caregiving functions provided in childhood are transposed onto romantic partners, identifying both risk and protective factors that contribute to relationship quality (Candel and Turliuc 2019; Velotti et al. 2023). Among these functions, the ability to regulate dyadic emotions plays a key role. Faccini et al. (2023) recently proposed the concept of dyadic emotion regulation, emphasising the couple context as a critical space for implementing strategies to regulate both one's own and the partner's emotions.

Given the importance of these functions, increasing attention has been directed toward psychopathological conditions that may affect both individual and relational well‐being (Kabiri et al. 2017; Fávero et al. 2021; Cataudella et al. 2023), including excessive dependence on a romantic partner. Love Addiction, first introduced in the literature nearly 50 years ago (Peele and Brodsky 1975), remains a poorly defined condition, with unclear underlying mechanisms. To address this gap, the present research aimed to investigate the psychopathological mechanisms underlying LA, within the broader dynamics of romantic relationships. The goal of this study was to explore love addiction through a relational framework, potentially differentiating it from behavioural addictions—the diagnostic category under which it has traditionally been conceptualised. To this end two studies were conducted, adopting attachment theory and dyadic emotion regulation as guiding theoretical frameworks.

The first study's findings highlighted a significant positive association between all subscales of anxious attachment and love addiction inventory (LAI), controlling for age and gender, aligning with previous literature (Rogier et al. 2024). Furthermore, a positive correlation with relationship obsessive‐compulsive disorder (ROCD) was observed, supporting Sussman's (2010) theoretical conceptualization. Specifically, the heightened doubts regarding partner interest that characterise love addiction were empirically confirmed. Interestingly, the absence of associations between LAI salience (orientation of feelings, thoughts, and behaviours toward the partner) and LAI mood modification (use of the partner for emotional regulation) with the subscales of the ROCI ‘Love for Partner’ and ‘Adequacy of Relationship’ is noteworthy. For individuals with love addiction, it is not their own feelings toward the partner or the relationship itself that are persistently questioned, but the reciprocity of the partner's feelings. This observation suggests that the obsessive preoccupation centers not on the relationship or the partner's feelings per se, but on validating the partner's emotional availability—leading to behaviours such as control tactics and constant reassurance‐seeking. This pattern is consistent with both the anxious attachment style (Mikulincer and Shaver 2016) and love addiction (Briggie and Briggie 2015). Moderation analyses further supported these conclusions, revealing that anxious attachment moderates the relationship between ROCD and Love Addiction. The interaction was stronger at higher levels of attachment insecurity, confirming the second hypothesis.

These results are also consistent with previous studies (Kabiri et al. 2017; Trak and Inozu 2019), which observed that anxious attachment was associated with the severity of ROCD symptoms. Finally, an additional result emerged. The interaction between anxious attachment and ROCD in relation to Love Addiction was weaker with increasing age. However, this is consistent with prior findings showing that attachment anxiety tends to decrease in middle adulthood compared to early adulthood (Chopik et al. 2013).

The second study aimed to examine whether difficulties in dyadic regulation are associated with the self‐defeating interpersonal style in relation to love addiction. Correlation analyses revealed that difficulties in dyadic regulation were associated with all LAI subscales, confirming the role of emotion regulation difficulties in love addiction. However, it was also found that the total LAI score was not associated with lack of awareness, but was positively associated with lack of dyadic clarity. These findings were confirmed through moderation analyses, where only the interaction between lack of dyadic clarity and the self‐defeating interpersonal style was significant in relation to love addiction, partially confirming our third and fourth hypotheses.

Thus, the ability to clearly understand one's emotions (emotional clarity), rather than simply being attentive to them (emotional awareness), appears to play a crucial role. Although the involvement of both abilities was hypothesised, the literature suggests that emotional clarity is a stronger predictor of psychopathological symptoms than other emotion regulation strategies or alexithymia (Pugach and Wisco 2023). According to these authors, low emotional clarity is associated with greater reliance on disengagement strategies in the face of emotional material, such as trauma cues, particularly among individuals with PTSD. Consistent with this perspective, it can be hypothesised that due to difficulties in emotional clarity, individuals detach more from emotional content associated with relational maltreatment, facilitating excessive dependence on the partner despite experiencing suffering. Moreover, our results showed that the interaction between lack of dyadic clarity and self‐defeating interpersonal style became stronger as emotional dysregulation increased. Although the literature on this topic is still emerging, it is plausible to hypothesise that in individuals with a self‐defeating interpersonal style, significant difficulties in emotional clarity may promote emotional disengagement from abusive relational stimuli, reinforcing maladaptive attitudes aimed at preserving the relationship at all costs, and thereby promoting excessive dependence on the partner.

## 8 Conclusion, Implication, and Limits

The aim of the present study was to investigate the role of dimensions associated with the clinical picture of love addiction. The core characteristics of LA—including prioritisation of the romantic relationship for individual well‐being and the orientation of thoughts and behaviours toward the partner—suggest the need to conceptualise the study of Love Addiction within a relational theoretical framework.

To this end, we conducted two studies investigating the association between LA and variables linked to dysfunctional couple dynamics, specifically relationship obsessive‐compulsive disorder and self‐defeating interpersonal style. These investigations were grounded in two major frameworks traditionally employed in the study of romantic bonds: attachment theory and emotion regulation. In the first study, a significant interaction between ROCD symptoms and an anxious attachment style was found in the association with Love Addiction. In the second study, the moderating role of lack of dyadic clarity was identified, reinforcing the importance of dyadic emotion regulation processes.

These preliminary results expand current knowledge on the construct of love addiction. From both clinical and empirical perspectives, our findings highlight the potential for differentiating love addiction from the broader diagnostic cluster of behavioural addictions by emphasising the critical role of relational functioning processes. Unlike other behavioural addictions, in love addiction, the psychological processes underpinning relational functioning appear to be central. For individuals with LA, the relationship with their romantic partner becomes a primary source of emotional regulation and self‐esteem maintenance. As proposed by Sanches and John (2018), a key feature of LA is the progressive development of dependency on the partner to the extent that the individual perceives them as essential for their emotional and psychological well‐being.

Despite the promising findings, several limitations must be acknowledged. First, our sample was not balanced between clinical and community populations, suggesting the need to replicate these results with a more balanced or fully clinical sample to ensure greater generalisability.

Second, in assessing love addiction, we utilised the Love Addiction Inventory (Costa et al. 2021). However, given the observed results, it is possible that this instrument may not fully capture all the nuanced facets of the construct. Future research should consider developing new assessment tools, ideally integrating clinical observations and empirical findings, to better reflect the complexity of love addiction.

Future studies should also seek to deepen and broaden these preliminary results, proposing a more refined diagnostic conceptualisation of love addiction. Building theoretical models that explicitly integrate relational functioning, attachment patterns, and dyadic emotion regulation could contribute to a more precise understanding of this emerging clinical phenomenon.

## Ethics Statement

All procedures performed in studies involving human participants were in accordance with the ethical standards of the institutional and/or national research committee and with the 1964 Helsinki Declaration and its later amendments or comparable ethical standards. This study was approved by the Ethics Committee of the University of (anonymized for review) (Research project approval N. 0078 del 24 gennaio 2022).

## Consent

Informed consent was obtained from all individual participants included in the study. The current research procedure was approved by the Ethical Board of Sapienza University of Rome (Blinded for review N. 0078/22).

## Conflicts of Interest

The authors declare no conflicts of interest.

## Data Availability

The data that support the findings of this study are available on request from the corresponding author. Authors did not preregister this research in an independent, institutional registry.

## References

[ijop70079-bib-0001] Ambler, K. , K. V. Petrides , and P. A. Vernon . 2023. “Relations Between a Self‐Defeating Interpersonal Style and Trait Emotional Intelligence.” Personality and Individual Differences 203: 112026. 10.1016/j.paid.2022.112026.

[ijop70079-bib-0038] Arbinaga, F. , M. I. Mendoza‐Sierra , B. M. Caraballo‐Aguilar , et al. 2021. “Jealousy, Violence, and Sexual Ambivalence in Adolescent Students According to Emotional Dependency in the Couple Relationship.” Children 8: 993. 10.3390/children8110993. 34828706 PMC8623033

[ijop70079-bib-0002] Atkinson, B. E. , and P. A. Vernon . 2017. “The SELF‐DISS: A Comprehensive Measure of Self‐Defeating Interpersonal Style.” Electronic Thesis and Dissertation Repository. 4896. https://ir.lib.uwo.ca/etd/4896.

[ijop70079-bib-0039] Briggie, A. , and C. Briggie . 2015. “Love Addiction: What's Love Got to Do With It?” In The behavioral addictions, edited by M. S. Ascher and P. Levounis , 153–172. American Psychiatric Publishing.

[ijop70079-bib-0003] Candel, O. S. , and M. N. Turliuc . 2019. “Insecure Attachment and Relationship Satisfaction: A Meta‐Analysis of Actor and Partner Associations.” Personality and Individual Differences 147: 190–199. 10.1016/j.paid.2019.04.037.

[ijop70079-bib-0004] Cataudella, S. , G. Rogier , S. Beomonte Zobel , and P. Velotti . 2023. “The Relation of Anxiety and Avoidance Dimensions of Attachment to Intimate Partner Violence: A Meta‐Analysis About Victims.” Trauma, Violence & Abuse 24: 1047–1062. 10.1177/15248380211050595. 34779309

[ijop70079-bib-0005] Cavalli, R. G. , ILARG Consortium , C. Tacchino , and P. Velotti . 2024. “Understanding Psychological and Psychopathological Facets in Love Addiction: Preliminary Results.” Sexual Health & Compulsivity 32: 1–24. 10.1080/26929953.2024.2392202.

[ijop70079-bib-0006] Chopik, W. J. , R. S. Edelstein , and R. C. Fraly . 2013. “From the Cradle to the Grave: Age Differences in Attachment From Early Adulthood to Old Age.” Journal of Personality 81, no. 2: 171–183. 10.1111/j.1467-6494.2012.00793.x. 22583036

[ijop70079-bib-0007] Costa, S. , M. Ingrassia , N. Barberis , M. D. Griffiths , and L. Benedetto . 2021. “The Love Addiction Inventory: Preliminary Finding of the Development Process and Psychometric Characteristic.” International Journal of Mental Health and Addiction 19: 651–668. 10.1007/s11469-019-00097-y.

[ijop70079-bib-0008] DeWall, C. N. , J. K. Maner , T. Deckman , and D. A. Rouby . 2011. “Forbidden Fruit: Inattention to Attractive Alternatives Provokes Implicit Relationship Reactance.” Journal of Personality and Social Psychology 100: 621–629. 10.1037/a0021749. 21244177

[ijop70079-bib-0009] Doron, G. , D. Derby , O. Szepsenwol , and D. Talmor . 2012. “Flaws and all: Exploring Partner‐Focused Obsessive‐Compulsive Symptoms in Two Non‐Clinical Cohorts.” Journal of Obsessive‐Compulsive and Related Disorders 1, no. 4: 234–243. 10.1016/j.jocrd.2012.05.004.

[ijop70079-bib-0032] Earp, B. D. , O. A. Wudarczyk , B. Foddy , and J. Savulescu . 2017. “Addicted to Love: What is Love Addiction and When Should it be Treated?” Philosophy, psychiatry, & psychology: PPP 24, no. 1: 77–92. 10.1353/ppp.2017.0011. PMC5378292 28381923

[ijop70079-bib-0010] Faccini, F. , G. Rogier , R. G. Cavalli , A. Santona , and P. Velotti . 2023. “The Difficulties in Emotion Regulation Scale–Dyadic Version: A New Tool for the Evaluation of the Dyadic Dysregulation in Couple Relationships.” Family Process 63, no. 3: 1231–1248. 10.1111/famp,12956. 38263528

[ijop70079-bib-0011] Fávero, M. , L. Lemos , D. Moreira , F. N. Ribeiro , and V. Sousa‐Gomes . 2021. “Romantic Attachment and Difficulties in Emotion Regulation on Dyadic Adjustment: A Comprehensive Literature Review.” Frontiers in Psychology 12: 723823. 10.3389/fpsyg.2021.723823. 34966317 PMC8710590

[ijop70079-bib-0040] Gioia, F. , V. Rega , and V. Boursier . 2021. “Problematic Internet Use and Emotional Dysregulation Among Young People: A Literature Review.” Clinical neuropsychiatry 18, no. 1: 41–54. 10.36131/cnfioritieditore20210104. 34909019 PMC8629046

[ijop70079-bib-0012] Gómez‐López, M. , C. Viejo , and R. Ortega‐Ruiz . 2019. “Well‐Being and Romantic Relationships: A Systematic Review in Adolescence and Emerging Adulthood.” International Journal of Environmental Research and Public Health 16, no. 13: 2415. 10.3390/ijerph16132415. 31284670 PMC6650954

[ijop70079-bib-0013] Gratz, K. L. , and L. Roemer . 2004. “Multidimensional Assessment of Emotion Regulation and Dysregulation: Development, Factor Structure, and Initial Validation of the Difficulties in Emotion Regulation Scale.” Journal of Psychopathology and Behavioral Assessment 26, no. 1: 41–54. 10.1023/B:JOBA.0000007455.08539.94.

[ijop70079-bib-0014] Griffiths, M. 2005. “A ‘Components’ Model of Addiction Within a Biopsychosocial Framework.” Journal of Substance Use 10, no. 4: 191–197. 10.1080/14659890500114359.

[ijop70079-bib-0041] Hayes, A. F. 2022. Introduction to Mediation, Moderation, and Conditional Process Analysis: A Regression‐Based Approach. 3rd ed. Guilford Press.

[ijop70079-bib-0033] Hazan, C. , and P. Shaver . 1987. “Romantic Love Conceptualized as an Attachment Process.” Journal of Personality and Social Psychology 52, no. 3: 511–524. 10.1037/0022-3514.52.3.511. 3572722

[ijop70079-bib-0015] Kabiri, M. , H. T. Neshat‐Doost , and H. A. Mehrabi . 2017. “The Mediating Role of Relationship Obsessive‐Compulsive Disorder in Relation to Attachment Styles and Marital Quality in Women.” Journal of Research and Health 7: 1065–1073. 10.18869/acadpub.jrh.7.5.1065.

[ijop70079-bib-0016] Kazan, D. , A. L. Calear , and P. J. Batterham . 2016. “The Impact of Intimate Partner Relationships on Suicidal Thoughts and Behaviours: A Systematic Review.” Journal of Affective Disorders 190: 585–598. 10.1016/j.jad.2015.11.003. 26583348

[ijop70079-bib-0017] Melli, G. , C. Carraresi , A. Pinto , L. Caccico , and E. Micheli . 2018. “Valutare il disturbo ossessivo‐compulsivo da relazione: proprietà psicometriche della versione italiana di ROCI e PROCSI.” Italian Journal of Cognitive and Behavioural Psychotherapy 24, no. 3: 251–269.

[ijop70079-bib-0043] Mikulincer, M. , and P. R. Shaver . 2016. Attachment in Adulthood: Structure, Dynamics and Change. 2nd ed. Guilford Press.

[ijop70079-bib-0036] Mikulincer, M. , and P. R. Shaver . 2019. “Attachment Orientations and Emotion Regulation.” Current opinion in psychology 25: 6–10. 10.1016/j.copsyc.2018.02.006. 29494853

[ijop70079-bib-0018] Muñoz‐Rivas, M. , A. Bellot , I. Montorio , R. Ronzón‐Tirado , and N. Redondo . 2021. “Profiles of Emotion Regulation and Post‐Traumatic Stress Severity Among Female Victims of Intimate Partner Violence.” International Journal of Environmental Research and Public Health 18: 6865. 10.3390/ijerph18136865. 34206787 PMC8297086

[ijop70079-bib-0019] Murphy, G. C. M. , R. M. Horne , M. L. Visserman , and E. A. Impett . 2022. “Relationship Functioning Following a Large‐Scale Sacrifice: Perceived Partner Prosociality Buffers Attachment Insecurity.” Journal of Family Psychology 36, no. 6: 986–997. 10.1037/fam0000994. 35511555

[ijop70079-bib-0042] Peele, S. , and A. Brodsky . 1975. Love and Addiction. Broadrow.

[ijop70079-bib-0044] Pugach, C. P. , and B. E. Wisco . 2023. “Emotion Regulation Repertoires in Trauma‐Exposed College Students: Associations With PTSD Symptoms, Emotional Awareness, and Emotional Clarity.” Psychological trauma: theory, research, practice and policy 15, no. Suppl 1: S37–S46. 10.1037/tra0001200. 34843344

[ijop70079-bib-0021] Redcay, A. , and C. Simonetti . 2018. “Criteria for Love and Relationship Addiction: Distinguishing Love Addiction From Other Substance and Behavioral Addictions.” Sexual Addiction & Compulsivity 25, no. 1: 80–95. 10.1080/10720162.2017.1403984.

[ijop70079-bib-0020] Redcay, A. , and S. McMahon . 2021. “Assessment of Relationship Addiction.” Sexual and Relationship Therapy 36, no. 1: 116–125. 10.1080/14681994.2019.1602258.

[ijop70079-bib-0022] Reynaud, M. , L. Karila , L. Blecha , and A. Benyamina . 2010. “Is Love Passion an Addictive Disorder?” American Journal of Drug and Alcohol Abuse 36, no. 5: 261–267. 10.3109/00952990.2010.495183. 20545601

[ijop70079-bib-0031] Rick, J. L. , M. K. Falconier , and A. K. Wittenborn . 2017. “Emotion Regulation Dimensions and Relationship Satisfaction in Clinical Couples.” Personal Relationships 24, no. 4: 790–803. 10.1111/pere.12213.

[ijop70079-bib-0023] Rogier, G. , F. Di Marzio , C. Presicci , R. G. Cavalli , and P. Velotti . 2024. “Love Addiction and Sexual Satisfaction Within the Attachment Perspective: An Empirical Contribution.” Psychology & Sexuality 15: 694–709. 10.1080/19419899.2024.2334412.

[ijop70079-bib-0024] Sanches, M. , and V. P. John . 2018. “Treatment of Love Addiction: Current Status and Perspectives.” European Journal of Psychiatry 33, no. 1: 38–44. 10.1016/j.ejpsy.2018.07.002.

[ijop70079-bib-0025] Shaver, P. R. , M. Mikulincer , and J. Cassidy . 2019. “Attachment, Caregiving in Couple Relationships, and Prosocial Behavior in the Wider World.” Current Opinion in Psychology 25: 16–20. 10.1016/j.copsyc.2018.02.009. 29514115

[ijop70079-bib-0026] Shoop, J. N. 2019. “Individual Adjustment as a Predictor of Improvements in Romantic Relationship Quality From Adolescence to Adulthood.” Electronic Theses and Dissertation.

[ijop70079-bib-0037] Sussman, S. 2010. “Love Addiction: Definition, Etiology, Treatment.” Sexual Addiction & Compulsivity 17, no. 1: 31–45. 10.1080/10720161003604095.

[ijop70079-bib-0035] Thompson, R. A. 1994. “Emotion Regulation: A Theme in Search of Definition.” Monographs of the Society for Research in Child Development 59, no. 2‐3: 25–52. 10.2307/1166137. 7984164

[ijop70079-bib-0034] Trak, E. , and M. Inozu . 2019. “Developmental and Self‐Related Vulnerability Factors in Relationship‐Centered Obsessive Compulsive Disorder Symptoms: A Moderated Mediation Model.” Journal of Obsessive‐Compulsive and Related Disorders 21: 29–36. 10.1016/j.jocrd.2019.03.004.

[ijop70079-bib-0029] Velotti, P. , G. Rogier , R. Castellano , E. Glielmo , V. Alajmo , and G. C. Zavattini . 2023. “Cultural Adaptation and Validation of the Italian Version of the Current Relationship Interview.” Frontiers in Psychology 14: 1250471. 10.3389/fpsyg.2023.1250471. 37842691 PMC10572345

[ijop70079-bib-0028] Velotti, P. , G. Rogier , S. Beomonte Zobel , A. Chirumbolo , and G. C. Zavattini . 2022. “The Relation of Anxiety and Avoidance Dimensions of Attachment to Intimate Partner Violence: A Meta‐Analysis About Perpetrators.” Trauma Violence Abuse 23, no. 1: 196–212. 10.1177/1524838020933864. 32608337

[ijop70079-bib-0027] Velotti, P. , R. Castellano , and G. C. Zavattini . 2011. “Adjustment of Couples Following Childbirth: The Role of Generalized and Specific States of Mind in an Italian Sample.” European Psychologist 16, no. 1: 1–10. 10.1027/1016-9040/a000022.

[ijop70079-bib-0030] Wei, M. , D. W. Russell , B. Mallinckrodt , and D. L. Vogel . 2007. “The Experiences in Close Relationship Scale (ECR)‐short Form: Reliability, Validity, and Factor Structure.” Journal of Personality Assessment 88, no. 2: 187–204. 10.1080/00223890701268041. 17437384

